# Markov model combined with MR diffusion tensor imaging for predicting the onset of Alzheimer’s disease

**DOI:** 10.1515/biol-2022-0714

**Published:** 2023-11-08

**Authors:** Lili Lang, Ying Wang

**Affiliations:** Basic Medical College, Changzhi Medical College, Changzhi, Shanxi, 046000, China; Endoscopic Chamber, Muling Town Forest District Hospital, Mudanjiang, Heilongjiang, 157513, China

**Keywords:** Alzheimer’s disease, magnetic resonance, diffusion tensor imaging, white matter, biomarker

## Abstract

Alzheimer’s disease (AD) affects cognition, behavior, and memory of brain. It causes 60–80% of dementia cases. Cross-sectional imaging investigations of AD show that magnetic resonance (MR) with diffusion tensor image (DTI)-detected lesion locations in AD patients are heterogeneous and distributed across the imaging area. This study suggested that Markov model (MM) combined with MR-DTI (MM + MR-DTI) was offered as a method for predicting the onset of AD. In 120 subjects (normal controls [NCs], amnestic mild cognitive impairment [aMCI] patients, and AD patients) from a discovery dataset and 122 subjects (NCs, aMCI, and AD) from a replicated dataset, we used them to evaluate the white matter (WM) integrity and abnormalities. We did this by using automated fiber quantification, which allowed us to identify 20 central WM tracts. Point-wise alterations in WM tracts were shown using discovery and replication datasets. The statistical analysis revealed a substantial correlation between microstructural WM alterations and output in the patient groups and cognitive performance, suggesting that this may be a potential biomarker for AD. The MR-based classifier demonstrated the following performance levels for the basis classifiers, with DTI achieving the lowest performance. The following outcomes were seen in MM + MR-DTI using multimodal techniques when combining two modalities. Finally, a combination of every imaging method produced results with an accuracy of 98%, a specificity of 97%, and a sensitivity of 99%. In summary, DTI performs better when paired with structural MR, despite its relatively weak performance when used alone. These findings support the idea that WM modifications play a significant role in AD.

## Graphical abstract

**Figure:**
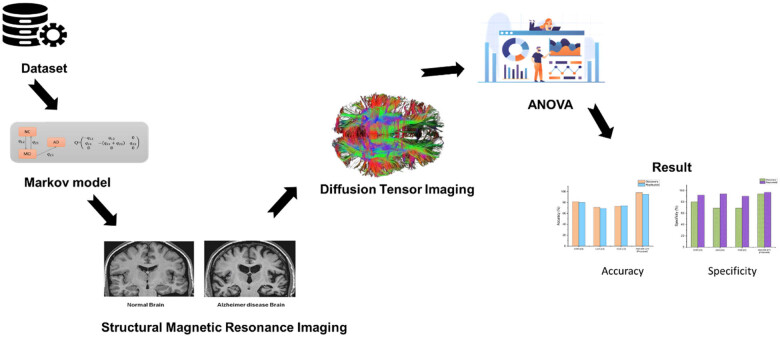

## 1 Introduction

**Figure 1:**
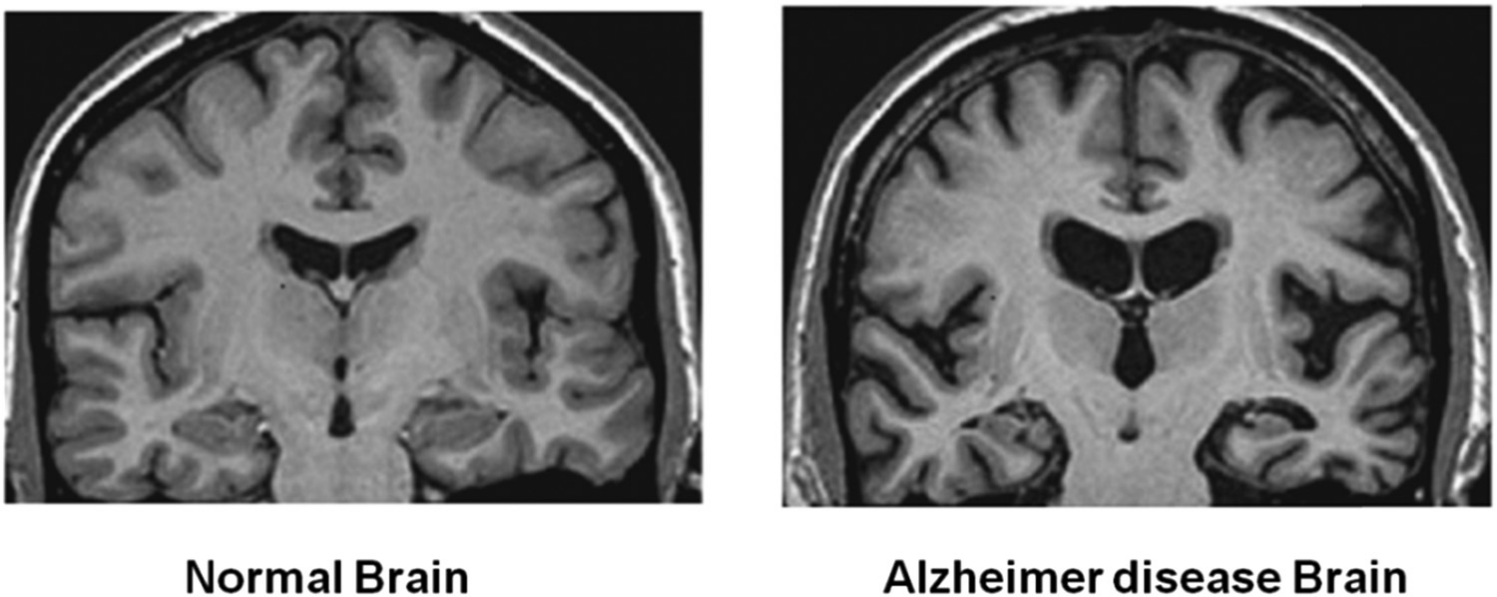
MR-DTI of ordinary and Alzheimer’s brain.

Alzheimer’s disease (AD) is a common neurological condition that causes gradual and permanent memory loss, impacting mental and social functioning. Since moderate memory issues are a hallmark of the early stages of AD, we often try to pinpoint the most telling signs of this illness to enhance our ability to diagnose it early and start treating it with customized drug regimens. Furthermore, while amnestic onset is the most common form of AD, it is vital to remember that atypical onsets occur in roughly 20% of cases [1]. Although there is a wide range of cognitive problems in the early clinical symptoms of AD, these abnormalities may be linked to deterioration in select particular brain areas. Initially, AD often exhibits memory loss and poor judgment. The patient’s dependence increases as the disease worsens, and constant care is needed in the last stages. Although there is currently no cure for AD, some drugs may temporarily alleviate symptoms, slowing the progression of the illness and, therefore, delaying the stage when the patient becomes entirely reliant [2]. The “Alzheimer’s Disease Neuroimaging Initiative (ADNI),” currently in its 17th year and fourth phase, aims to enhance clinical trials for AD-modifying or AD-preventive drugs by using its expanding collection of data and samples, which are made accessible to researchers all over the globe. To do this, we combined through all the studies that have used ADNI data and models as of the end of 2017; this assessment updates past research. We examine how ADNI has advanced our knowledge of the course of the illness in AD and how this information might be used in productive clinical trials that result in the approval of drugs that can delay the onset of AD and eventually prevent it [3]. New MRI techniques have been developed recently for monitoring water exchange across the blood–brain barrier (BBB). These techniques provide a fresh way to evaluate the porosity of the BBB without the need for contrast material. These contrast-agent-free MRI methods primarily use two distinct forms. One uses diffusion after labeling intravascular water using the arterial spin labeling method, multiple echoes, or phase contrast techniques to observe how the marked water changes dynamically in intravascular and extravascular areas. The second method makes use of “filter-exchange imaging (FEXI),” a technique modified for clinical use from “diffusion exchange spectroscopy.” By looking into the “intravoxel incoherent motion” of capillary water, creating a suitable “diffusion weighting to specifically filter out intravascular water, and then quantitatively monitoring the water exchange between intra- and extravascular space via the second diffusion encoding,” water exchange across the BBB in humans may be measured using FEXI. Recent research demonstrated that water exchange across the BBB is a more reliable biomarker than the more frequent diagnostic of contrast agent leakage from the BBB in diagnosing moderate BBB breakdown in an AD rat model. Even though the specific chemical pathway for water exchange across the BBB is yet understood, the fact that water molecules are smaller than MRI contrast chemicals may make them more prone to BBB leakage, water-exchange-based magnetic resonance imaging (MRI) may detect BBB leakage in individuals with AD [4]. Figure 1 depicts the MR-DTI of a normal brain and a brain with Alzheimer’s disease.

Cognitive impairment that people perceive without any clinical indication on the findings of neurocognitive examination is referred to as emotional and cognitive deficits (SCD). The SCD phase often occurs in older persons with dementia syndromes before the illness manifests itself. Although growing interests and mounting data show a link between SCD and its advancement to MCI and AD, there are a variety of views on how to classify this stage and its importance. Since older people with normal controls (NC) may also acquire SCD due to normal aging, it is still up to question how to discriminate between disease progression types in them. An increasing number of researchers are studying neuroimaging data and biomarkers to understand the underlying processes and features of SCD. Their objective is to illustrate how these characteristics might predict future cognitive impairment and perhaps intervene to reduce or reverse the advancement of the condition. [5]. AD has crippling impacts on individuals, their families, and society, and it causes caregivers enormous suffering. Between cognitively normal and dementia, MCI is a stage. The percentage of MCI patients that convert to AD ranges from 10 to 20% during the first year, with the remaining participants either developing other types of dementia or staying stable. The correct treatment of AD patients requires early detection before the onset of severe symptoms, which is still problematic. The ability to track the disease’s course might 1 day allow for the early diagnosis of Alzheimer’s and the subsequent medication administration to prevent the progression of disease. Patients with MCI share various cognitive traits, making it challenging to distinguish between those with stable MCI and those with progressive MCI. When put together, these two challenges make it much more challenging for any method of detecting AD progression to distinguish between cognitive normal CN, MCI, and AD [6].

Diffusion MRI (dMRI) has been used to identify brain structural impairments compared to controls to explain well the established cognitive deterioration in hemodialysis (HD) patients. The mean apparent propagator model was analyzed in the study by Richerson et al. [7] to understand diffusion in HD patients. Soussia and Rekik [8] found that it is now feasible to single out specific brain regions and trace how they evolved. They found that most resources divided MCI and AD into discrete categories. In the study by Gronemann et al. [9], the primary explanations for this low reproducibility are the use of potentially inaccurate diffusion tensor image (DTI) parameters produced from the single-tensor model and the small sample size. In research [10], the segmentation of brain tissue and identifying schizophrenia in gray matter are problems covered in this study. In the study by Minosse et al. [11], 35 patients who tested positive for the human immunodeficiency virus and 20 healthy controls were compared microstructurally using the neurite orientation dispersion and density imaging and DTI models. The three distinct *b*-values used to obtain diffusion-weighted images were 0, 1,000, and 2,500 s/mm^2^. In the study by Christidi et al. [12] that used a standardized neuroimaging method to evaluate the connectivity between white and gray matter areas in the mesial temporal lobes, “amyotrophic lateral sclerosis” and healthy controls were contrasted. In AD, there are often described white matter (WM) microstructural abnormalities. The most remarkable WM abnormalities in presymptomatic AD are found in certain brain parts, but it is unknown what molecular mechanisms cause WM abnormality as the illness progresses [13]. There were substantial gains in cardiorespiratory fitness, cognitive ability, and MRI findings among 46 healthy older persons in a study [14] that evaluated the advantages of a 12-week aerobic training program to a waitlist control condition. According to traditional DTI investigations and research that has already been verified, high-gradient dMRI is considered sensitive to the axonal substrate of age-related WM degeneration [15]. The goal of the study by Shen et al. [16] was to connect midlife risk factors, early DTI-measured WM abnormalities, and age and gender to understand the intricate interplay between these factors. While it has not been researched how “moderate-to-vigorous physical activity” affects AD development in individuals with Down syndrome, it has been shown to promote cognitive performance and protect against age-related alterations in brain structure and function in usually growing persons [17]. In this work, the dMRI studies in DLB cohorts that were compared to dementia in Parkinson’s disease (PDD), AD, or healthy controls are evaluated [18]. To ascertain if autopsy-confirmed “progressive supranuclear palsy (PSP) with Richardson’s syndrome (PSP-RS)” and “PSP with predominant speech/language disability (PSP-SL)” can be distinguished using “diffusion tractography of the SCP and dentatorubrothalamic tract” [19]. Herdick et al. [20] examined the changes in cholinergic basal forebrain volume, mean diffusivity, and functional connectivity throughout the spectrum of AD. They also evaluated how these alterations were impacted by amyloid pathology. As a result, we used a Markov model (MM) in conjunction with MR-DTI to forecast the start of AD.

Contributions of this study

- This study suggested that MM combined with MR-DTI (MM + MR-DTI) was offered as a method for predicting the onset of AD.
- The selected datasets were used to evaluate the WM integrity and abnormalities.
- Using automated fiber quantification (AFQ), we can identify 20 central WM tracts. Point-wise alterations in WM tracts were shown using discovery and replication datasets.
- The MR-based classifier demonstrated the following performance levels for the basis classifiers, with DTI achieving the lowest performance.

## 2 Material and methods

### 2.1 Data collection

The medical ethics committee of the Chinese PLA General Hospital gave its stamp of approval to this research [21]. Each participant or their guardian signed a written informed consent document. Patients with neurological conditions who were either inpatients or outpatients at the “Chinese PLA General Hospital” ranged in age from 55 to 85. The “Chinese PLA General Hospital’s Department of Neurology” performed pre-MRI clinical, physical, and cognitive assessments.

### 2.2 Criteria for inclusion and exclusion

- Inclusion
  - AD patients’ inclusion
    - Presumptive AD diagnosis is based on the requirements for diagnosis set out by “The National Institute of Neurological and Communicative Disorders and Stroke” and the AD and “Related Disorders Association.”
    - A clinical data repository (CDR) between 1 and 2.
    - We are not using any nootropic medications, including cholinesterase inhibitors, at the moment.
    - Tolerance for MR scanning and competence in doing neuropsychological tests.
  - MCI patients’ inclusion
    - Six months or more of chronic memory trouble.
    - A CDR equal to 0.5.
    - A score of 26 or less on the ADL inventory, plus no signs of dementia.
    - Patients with amnestic mild cognitive impairment (aMCI) and AD as a result of AD satisfied the new diagnostic standards for these conditions.
  - NC patients’ inclusion
    - A healthy bodily condition.
    - A CDR is equal to zero.
    - No concerns about memory loss.
- Exclusion
  - Hypothyroidism, vitamin B12/folic acid deficiency, other metabolic disorders, and psychiatric disorders such as schizophrenia or depression.
  - MRI/computed tomography evidence of a stroke or a bleed in the brain.
  - Diseases of the neurological system that may affect cognitive abilities, such as PDD and epilepsy.
  - A previous history of stroke or other degenerative disease.

The patient possesses a metallic foreign body that would render an MR scan dangerous, such as a cochlear implant or a stent in the patient’s heart.

### 2.3 Image acquisition protocol

#### 2.3.1 MM

The “ADNI” data were used to design a multi-state MM and prediction framework that simulates AD progression. We analyzed ADNI data next. We grouped individuals by clinical diagnosis (NC, MCI, and AD) as the response variable. The Markov chain’s state change was thought to be temporal in this study. Furthermore, we predicted that transition rates were not time-dependent but rather patient-specific, including regional volumetric data from MRI scans, cognitive evaluation scores, and demographics.

Let the illness condition of participant *j* at time *S*_*ji*_ be denoted by *T*_*ji*_ = *T*_*j*_(*s*_*ij*_). The *T*_*ji*_ for subject *j* is assumed to follow an n-state multi-state Markov process. A collection of states, *T* , is accepted. Let’s keep things simple and say *T* = {1, …, *m*}. If only *s*, *s*′ > 0 and *q*, *q*′ ∈ *T* , then the process is Markovian:

(1) \[O({T}_{j}(s,{s}^{^{\prime} })={q}^{^{\prime} }{\mathrm{|}}{T}_{j}(d)=q,{{\mathscr{F}}}_{w},w\lt s)=O({T}_{j}(s,{s}^{^{\prime} })={q}^{^{\prime} }{\mathrm{|}}{T}_{j}(d)=q),]\]

where *F*_*w*_ represents the whole set of observations made up to time *w* for the Markov process, i.e., given the past events of the procedure up to the current time *s*, the state of the activity at another point in time *s*, *s*′ relies exclusively on the state of the present time *s*. The transition probability matrix between the conditions of a multi-state Markov process is a way to characterize the process and is related to the initial distribution of the process at time 0.

As a replacement, we may define *Q*(*s*) = ((*oqq*′(*s*, *s* + *s*′))) as the matrices of transition rates:

(2) \[{oq}{q}^{^{\prime} (s)}=\mathop{\mathrm{lim}}\limits_{{s}^{^{\prime} }\to 0}\left[\frac{O({T}_{j}(s,{s}^{^{\prime} })={q}^{^{\prime} }|{T}_{j}(d)=q)}{{q}^{^{\prime} }}\right].]\]

**Figure 2:**
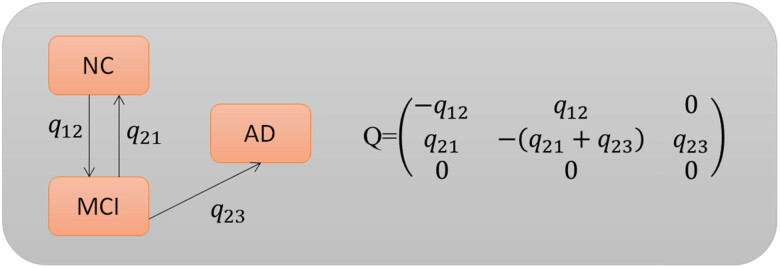
Transition rate matrix.

The immediate risk of changing from stage *q* to stage *q*′ is also known as transition intensity or *oqq*′ *Q* is the definition of our transition rate matrix in Figure 2. After entering the AD stage, the model believes there is no way to return to the previous stage. The transition patterns are shown in Figure 2. There are not enough data to estimate the parameters involved in the transitions from NC to AD and MCI.

According to equation (2), the intensity matrix *Q* may be considered the derivative of the matrix *o*, which represents the probabilities of transitions. For each *q* ∈ *s*, the corresponding rows in *Q* are 0; therefore, \[{oq}{q}^{^{\prime} }(s)=-{\sum }_{{q\text{'}}\ne q}{rqq\text{'}}(s)]\] . For an isotropic Markov chain in continuous time, we prove that *r*_0,*qq*_′ exp(*sQ*).

Take the collection of variables that explain at time *s* to be *V*(*s*). We then use this to describe the intensity matrix *Q*:

(3) \[{oq}{q}^{^{\prime} }(s,V(s))={r}_{0,{qq}^{\prime} }\exp ({\beta }_{{qq}^{\prime} }^{S}V(s)),]\]

where*V*(*s*) is an explanatory variable for *q* ≠, and *β*, and *qq*′ is a collection of regression parameters. In this work, we refer to *V*(*s*) as patient-specific variables.

The changeover time is undetermined but within a particular time range. This entire means that transition periods between states are suppressed at intervals. As a result, we may deduce the following about topic *j* likelihood:

(4) \[{K}_{j}=O({T}_{j1},\ldots ,{T}_{{jxj}})=O({T}_{j1})\mathop{\prod }\limits_{i=2}^{{x}_{j}}O({T}_{j1}{\mathrm{|}}{T}_{{ji}-1}){{ot}}_{j}({s}_{j1})\mathop{\prod }\limits_{i=2}^{{x}_{j}}{{ot}}_{j}({s}_{i-1}){T}_{j}({s}_{i})({s}_{j}-{s}_{i-1}).]\]

Evaluation at *s* = *s*_*j*_ − *s*_*i*−1_ in the transition probability matrix *o*(*T*)*f* at the (*s*_*i*−1_) row and *T*_*j*_(*s*_*i*_) column if the result is ot_*j*_(*s*_*i*−1_)*T*_*j*_(*s*_*i*_)(*s*_*j*_ − *s*_*i*−1_). The sum of all the times that subject *j* was seen is denoted by the variable *x*_*j*_. The event of interest in our model, as opposed to death, is the identification of MCI or potential AD, which sets it apart from illness-death models. Due to the small likelihood of a person with AD moving between the other stages, we consider it the last absorbing phase.

#### 2.3.2 Structural MRI

These imaging parameters were used for the T1 MPRAGE anatomic acquisitions: a “field of view of 256 mm × 256 mm” with a “matrix size of 256 mm × 256 mm”; 176 sagittal slices were acquired with a “voxel resolution of 1.0 mm × 1.0 mm × 3.0 mm” and a total time of acquisition (TA) of 6 min and 3 s.

#### 2.3.3 DTI

The following values were entered into the DTI software, using these parameters: TR = 7,800 ms, TE = 90 ms, NEX = 1, “matrix = 96 × 96 × 63 continuous axial slices,” “voxel size = 2 × 2 × 2 mm^3^,” “frequency resolution = 1,628 Hz/pixel,” “echo spacing = 0.72 ms,” and several excitations = 1. Sixty-three non-collinear orientations were scanned 62 times each at a diffusion weighting value of 1,000,000 s/mm^2^. Using “Generalized Autocalibrating Partially Parallel Acquisitions,” the scanning time was reduced to around 9 min.

### 2.4 Statistical analysis

Local changes associated with AD may be evaluated by comparing tract profiles retrieved throughout the whole WM tract. We analyzed 100 locations along each fiber tract for group-level diffusion metrics. After adjusting for age and gender, we used a one-way analysis of variance (ANOVA) on the point-by-point diffusion data to test for statistically significant differences between groups for each fiber tract. Post hoc analysis using two-sample two-sided *t*-tests showed no effect of age or gender on statistically significant differences (*P* < 0.05) across groups.

#### 2.4.1 ANOVA

ANOVA is a statistical method for breaking down aggregated reports of experimental variability into individual components. When there are three or more datasets, the association between the variables has been determined using a one-way ANOVA. ANOVA is the traditional analysis of variance. The F-statistic measures how often the null model, based on the anthropocentric idea, has average sums of squares. The least-squares approach calculates the parameters on the presumption that all variances are equal. One way to put it would be:

(5) \[S={\mathrm{M}}{{\mathrm{H}}}_{{\mathrm{between}}}/{\mathrm{M}}{{\mathrm{H}}}_{{\mathrm{error}}},]\]

where

(6) \[{\mathrm{M}}{{\mathrm{H}}}_{{\mathrm{between}}}=\frac{{\sum }_{i=1}^{k}l{({\bar{x}}_{i}-\bar{x})}^{2}}{n-1},]\]

and

(7) \[{\mathrm{M}}{{\mathrm{H}}}_{{\mathrm{error}}}=\frac{{\sum }_{i=1}^{k}{\sum }_{j=1}^{{l}_{i}}{({x}_{{ij}}{-x}_{i})}^{2}}{l-k}.]\]

The Welch test statistic is defined as:

(8) \[Y=\frac{{\sum }_{i=1}^{k}{y}_{j}{[}{({x}_{i}-\mathop{x}\limits^{\sim })}^{2}/(K\left-1)]}{1+\frac{2(k\left-2)}{{k}^{2}-1}{\sum }_{i=1}^{k}\left[{\left(,1-\frac{{y}_{j}}{u}\right)}^{2}/({l}_{i}\left-1)\right]},]\]

where \[{l}_{i}=\frac{n}{{t}_{1}^{2}}]\] , \[{u}={\sum }_{i=1}^{k}{l}_{i},]\] and \[\dot{Y=\frac{1}{u}}{\sum }_{i=1}^{k}{l}_{i}{y}_{j}]\] is defined as:

(9) \[s=\frac{{k}^{2}-1}{3{\sum }_{i=1}^{k}\left[{\left(,1-\frac{{y}_{j}}{u}\right)}^{2}/({l}_{i}\left-1)\right]}.]\]

The definition of the Brown–Forsythe test statistic is defined as follows:

(10) \[{S}^{* }=\frac{{\sum }_{i=1}^{k}{o}_{j}{({\bar{x}}_{j}-\bar{x})}^{2}}{{\sum }_{i=1}^{k}(1-{l}_{i}/L){H}_{i}^{2}}.]\]

If *L* is true, then S* should be distributed according to a central *S* distribution with degrees of freedom *K* minus one and *s*, where *s* is defined as:

(11) \[1/s=\mathop{\sum }\limits_{i=1}^{k}{c}_{i}^{2}/({l}_{i}-1),{\mathrm{where}}\hspace{1em}{c}_{i}=\frac{\left(\phantom{\rule[-0.75em]{}{0ex}},\frac{1-{l}_{i}}{L}\right){H}_{i}^{2}}{{\sum }_{j=1}^{h}\left(\phantom{\rule[-0.75em]{}{0ex}},\frac{1-{l}_{i}}{L}\right){H}_{i}^{2}}.]\]

The new formula for computing the broader *S*-value is *S* = 1 – *l*_*i*_, where *s* is the measurement size:

(12) \[s=A\left({J}_{k-1l-k}\left(\frac{l-k}{k-1}{\mathop{t}\limits^{\sim }}_{b}\left(\frac{{l}_{1}{t}_{1}^{2}}{{B}_{1}{B}_{2},\ldots ,{B}_{k-1}},\frac{{l}_{2}{t}_{2}^{2}}{{B}_{1}{B}_{2},\ldots ,{B}_{K-1}},\hspace{1.5em}\frac{{l}_{3}{t}_{3}^{2}}{(1\left-{B}_{2}){B}_{3},\ldots ,{B}_{K-1}},\ldots \frac{{l}_{1}{t}_{k}^{2}}{(1\left-{B}_{k-1})}\right)\right)\right).]\]

The forecast concerns the independent beta stochastic processes in an F-distribution with parameters *k* = −*J* and *A* = kdof:

(13) \[{B}_{k}\sim {\mathrm{Beta}}\left(\mathop{\sum }\limits_{i=1}^{k}\frac{({l}_{i}\left-1)}{2},\frac{{l}_{k+1}-1}{2}\right),l=1,2,\ldots ,k-1.]\]

When working with beta random variables, the *P*-value is calculated using the *P*-value technique to calculate the anticipated value.


**Informed consent:** Informed consent has been obtained from all individuals included in this study.
**Ethical approval:** The research related to human use has been complied with all the relevant national regulations, institutional policies, and in accordance with the tenets of the Helsinki Declaration and has been approved by the Chinese PLA General Hospital.

## 3 Result

The suggested model [MM + MR + DTI] is activated in MATLAB/Simulink, and its effectiveness is contrasted with that of current models such as support vector machine (SVM), linear discriminant analyses (LDA), and extremely gradient boost (XGB). Accuracy, specificity, and sensitivity performance measures were examined using the proposed and current methodologies.

The tractography technique is needed to track many persons between two sites in the discovery or replication datasets. The ability to identify the left/right cingulum hippocampus fiber tracts was lacking in 62/32 of the 120 people in the discovery dataset and 88/65 of the 122 people in the replicated dataset. Consequently, the following investigations did not include these two fiber lines. Therefore, research was built based on the 18 major WM tracts of the total brain. There were 120 persons in the discovery dataset: 47 were diagnosed with AD, 34 with aMCI, and 39 were neurologically intact. Age and gender differences between any group comparisons were not statistically significant. To confirm the WM tract anomalies discovered and the classification performance, the duplicated dataset contained 37 aMCI patients, 42 AD patients, and 43 age- and gender-matched NCs. The Mini-Mental State Examination (MMSE) and other cognitive tests revealed a significant difference between the two groups, with NCs scoring higher and AD patients scoring worse in Table 1. Table 2 depicts the demographically based clinical and neuropsychological replicated data.

**Table 1:** Demographically based clinical and neuropsychological discovery data

Discovery dataset	aMCI	AD	NC	*P*-value
Number of subjects	34	47	39	
Sex (M/F)	13/21	20/27	18/21	0.792
\[{{\mathrm{AVLT}}-{\mathrm{delayed\; recall}}}^{{\mathrm{d}},{\mathrm{e}}}]\]	2.8 ± 2.3^a^	0.8 ± 101^a,b^	5.6 ± 2.0	<0.001
Age (year)	69.5 ± 8.8	69.7 ± 9.3	68.5 ± 7.1	0.796
AVLT – immediate recall^c,e^	4.4 ± 1.3^a^	2.1 ± 1.4^a,b^	5.8 ± 1.3	<0.001
AVLT – recongnition (new words)^e^	8.4 ± 1.5^a^	6.7 ± 3.4^a,b^	9.4 ± 1.1	<0.001
MMSE score	26.6 ± 2.5^a^	16.1 ± 7.3^a,b^	28.5 ± 1.4	<0.001
AVLT – recognition (primary words)^e^	9.0 ± 1.7^a^	6.8 ± 3.4^a,b^	9.8 ± 0.7	<0.001

a – Significant in comparison to NCs. b – Notable dissimilarity in comparison to those with a typical MCI. c – The average of the best three recalls scores. d – The results of a five-minute delayed recall test. e – Nineteen people with AD, two with aMCI, and one without dementia did not or would not finish the test.

**Table 2:** Demographically based clinical and neuropsychological replicated data

Replicated dataset	AD	NC	aMCI	*P*-value
Number of subjects	43	47	44	
Sex (M/F)	29/21	11/23	17/25	0.789
AVLT – delayed recall^c,e^	0.5 ± 5.1^a,b^	3.5 ± 1.1	1.7 ± 2.4^a^	<0.001
Age (year)	52.6 ± 8.1	66.4 ± 6.2	71.3 ± 9.1	0.851
AVLT – immediate recall^d,e^	2.1 ± 1.4^a,b^	6.7 ± 2.1	4.3 ± 2.4^a^	<0.001
AVLT – recognition (new words)^e^	5.4 ± 2.1^a,b^	8.4 ± 1.1	9.1 ± 1.4^a^	<0.001
MMSE score	18.1 ± 6.1	21.4 ± 3.5	21.5 ± 1.4	<0.001
AVLT – recongnition (primary words)^e^	5.9 ± 2.5^a,b^	5.1 ± 0.4	11.5 ± 8.5^a^	<0.001

a – Significant in comparison to NCs. b – Notable dissimilarity in comparison to those with a typical MCI. c – The average of the best three recalls scores. d – The results of a five-minute delayed recall test. e – Nineteen people with AD, two with aMCI, and one without dementia did not or would not finish the test.

One may determine a statement’s accuracy by dividing the total number of reports by the number of correct labels applied to those claims. This method’s accuracy depends on the classifier’s ability to distinguish between normal and abnormal brain images. Precision in statistics refers to:

(14) \[{\mathrm{Accuracy}}=\frac{({\mathrm{TP}}\left+{\mathrm{TN}})}{({\mathrm{TP}}+{\mathrm{TN}}+{\mathrm{FP}}\left+{\mathrm{FN}})},]\]

where, TP is the percentage of instances when aberrant MR-DTIs were recognized as such, FP s the total number of regular MR-DTI scans mislabeled as abnormal, TN is the percentage of regular MR-DTI scans that were properly diagnosed, and FN is the number of abnormal MR-DTI scans that were deemed normal.

**Figure 3:**
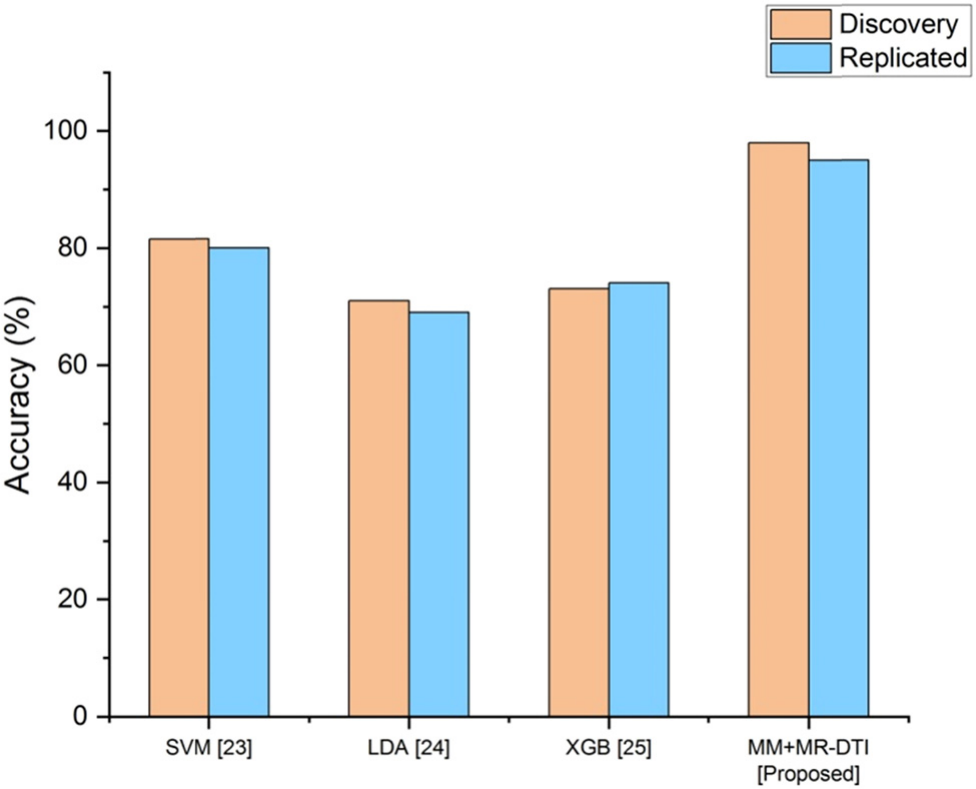
Comparison of the accuracy.

The accuracy comparison is shown in Figure 3. It was found that the suggested MM + MR-DTI are more precise than the currently used methods, which include SVM, LDA, and XGB. Table 3 depicts the performance analysis of the accuracy.

**Table 3:** Performance analysis of the accuracy

Methods	Accuracy (%)	
	Discovery	Replicated
SVM [23]	81.55	80
LDA [24]	71	69
XGB [25]	73	74
MM + MR-DTI [proposed]	98	95

Specificity measures a classifier’s skill in predicting true negatives. The method’s accuracy in detecting normal cases makes it stand out. The mathematical expression for this is as follows:

(15) \[{\mathrm{Specificity}}{\boldsymbol{=}}\frac{{\mathrm{TN}}}{{\mathrm{TN}}+{\mathrm{FP}}}.]\]

**Figure 4:**
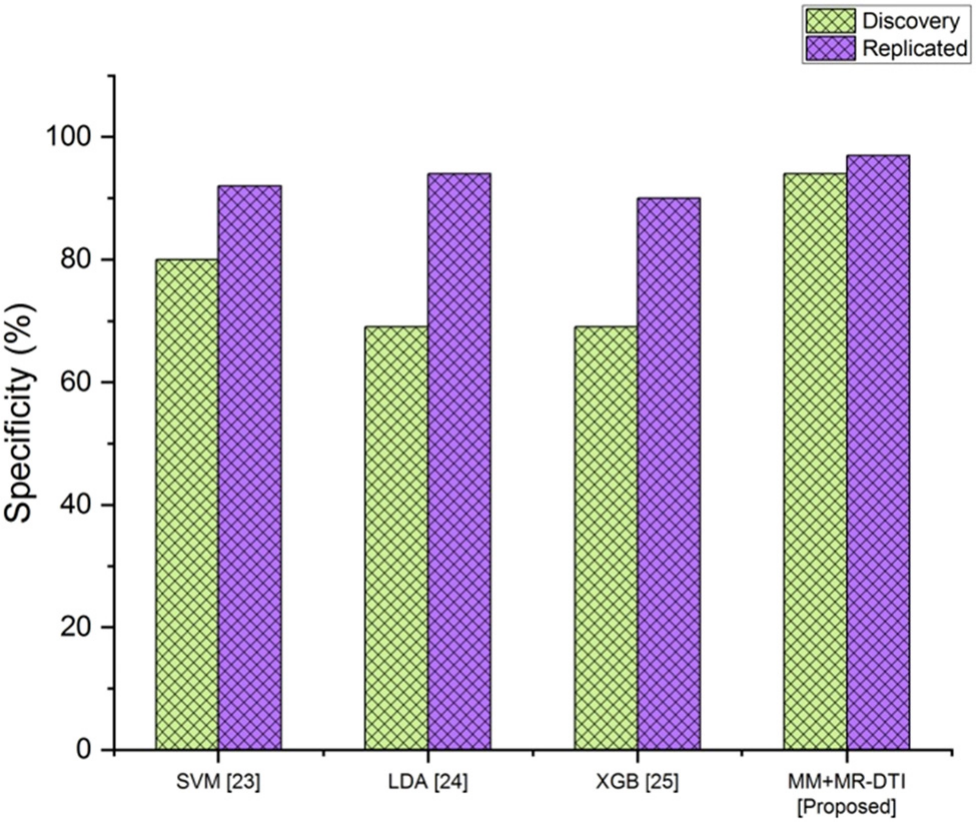
Comparison of the specificity.

The contrast in specificity is seen in Figure 4. Existing methods such as SVM, LDA, and XGB are compared to the proposed MM + MR-DTI in terms of specificity. Table 4 depicts the performance analysis of the specificity.

**Table 4:** Performance analysis of the specificity

Methods	Specificity (%)	
	Discovery	Replicated
SVM [23]	80	92
LDA [24]	69	94
XGB [25]	69	90
MM + MR-DTI [proposed]	94	97

When analyzing experimental findings, the word “sensitivity” refers to the percentage of samples that are expected to be positive. It mirrors what might happen if there were some excellent examples. The sensitivity is calculated using the following formula:

(16) \[{\mathrm{Sensitivity}}=\frac{{\mathrm{TP}}}{{\mathrm{TP}}+{\mathrm{FP}}}.]\]

**Figure 5:**
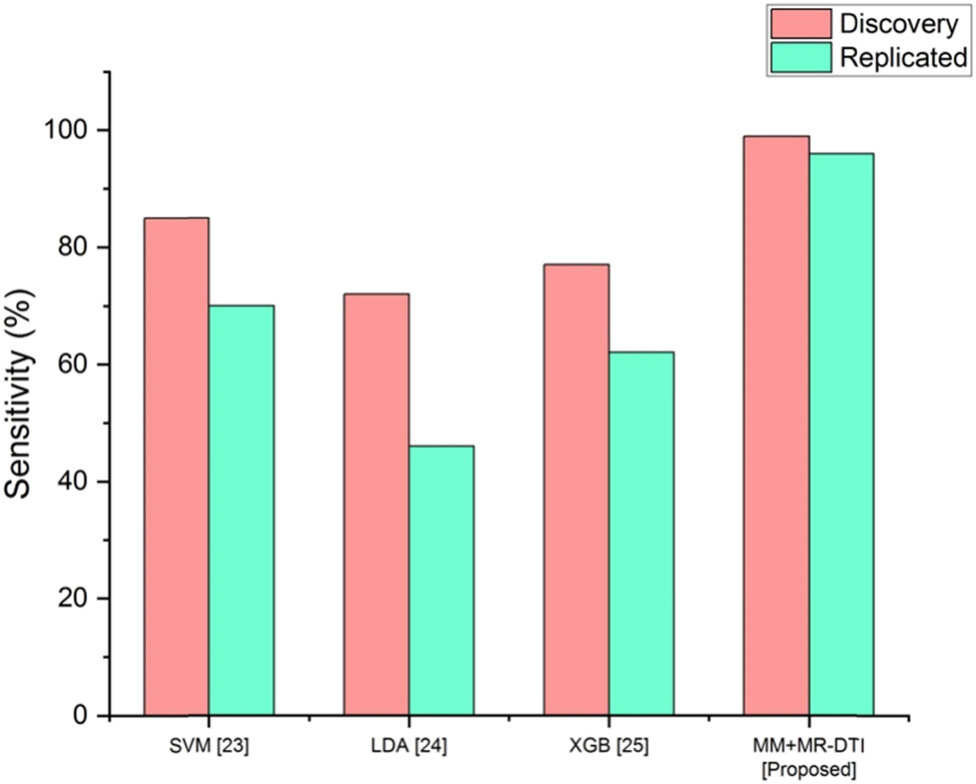
Comparison of the sensitivity.

The sensitivity comparison is shown in Figure 5. Compared to standard approaches such as SVM, LDA, and XGB, the specificity provided by the MM + MR-DTI methodology is much greater. Table 5 depicts the performance analysis of the sensitivity.

**Table 5:** Performance analysis of the specificity

Methods	Sensitivity (%)	
	Discovery	Replicated
SVM [23]	85	70
LDA [24]	72	46
XGB [25]	77	62
MM + MR-DTI [proposed]	99	96

**Figure 6:**
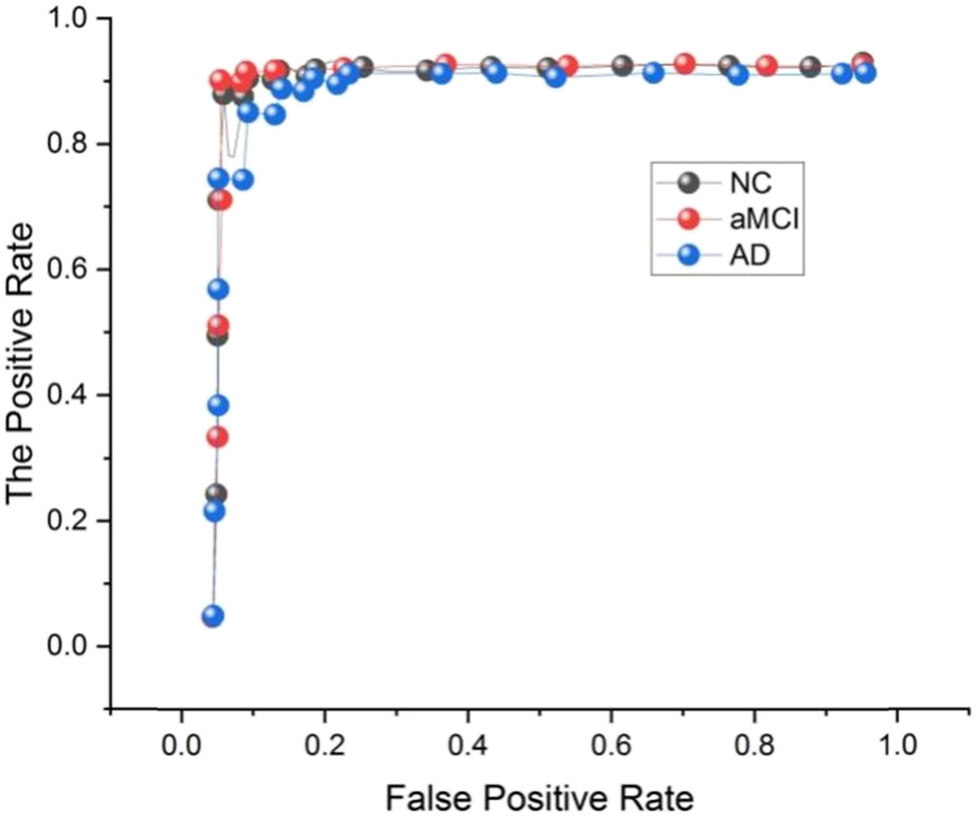
Comparison of the receiver-operating characteristic (ROC) curves for the MR-DTI.

**Figure 7:**
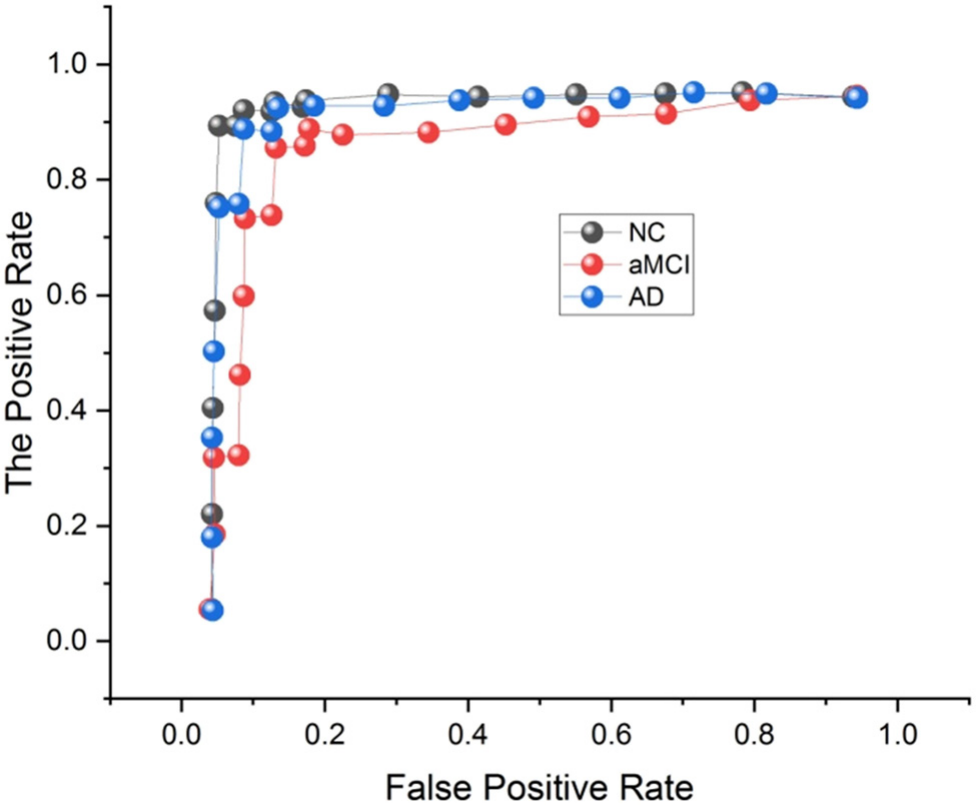
Comparison of the ROC curves for the MM + MR-DTI.

Figures 6 and 7 depict the ROC curve. At varying classification thresholds, the ROC curve contrasts the proportion of true positives (sensitivity) and false positives (specificity), respectively. When comparing the two rates, the actual positive rate is the percentage of really positive events adequately labeled as positive. In contrast, the false-positive rate is the percentage of genuinely adverse events wrongly labeled as positive. AUC-ROC is the area under the ROC curve and is a well-known performance statistic. It produces a single number that sums up a classifier’s total performance. AUC-ROC of 1 would represent an ideal classifier, whereas AUC-ROC of 0.5 would represent an unpredictable classifier. MM + MR-DTI’s higher AUC-ROC values generally indicate MR-DTI’s more excellent classification performance.

**Figure 8:**
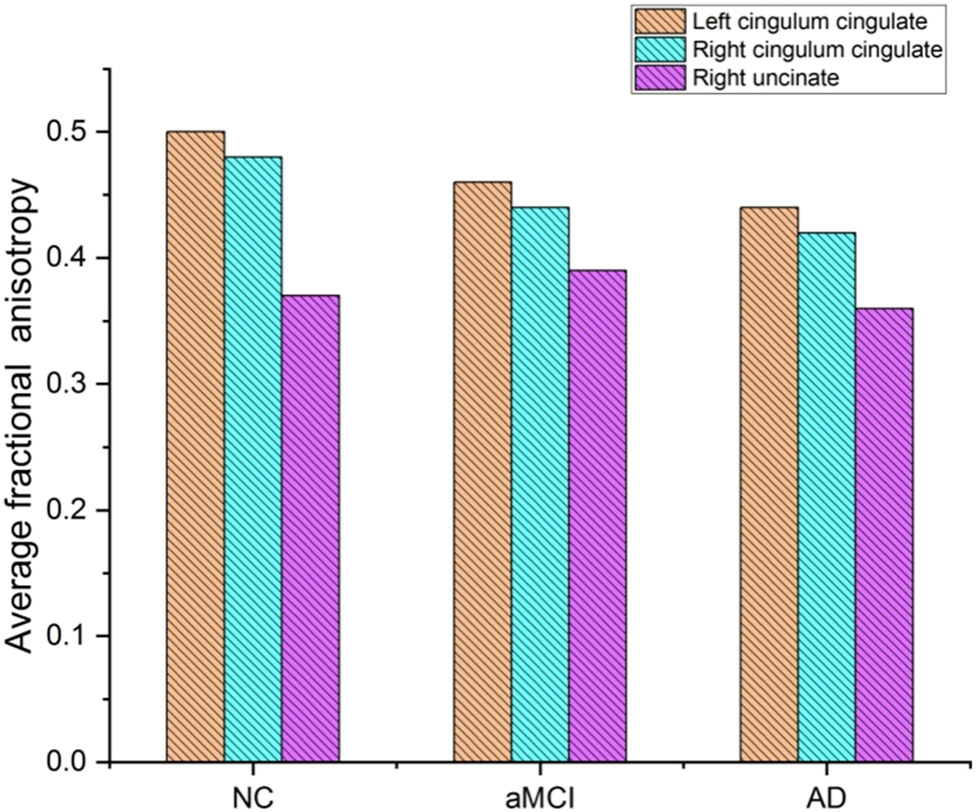
Average fractional anisotropy.

NC, aMCI, and AD patient groups’ fractional anisotropy (FA) profiles for the six identified fiber pathways are shown in Figure 8 – the mean tract FA profile for each group, plus or minus one standard error of the mean. The left cingulum cingulated, right cingulum cingulated, and right uncinate were shown to be significantly different between “NCs, aMCI patients, and AD” patients in the dataset. Compared to aMCI and AD, NC’s suggested method yields a much more significant average fraction anisotropy.

**Figure 9:**
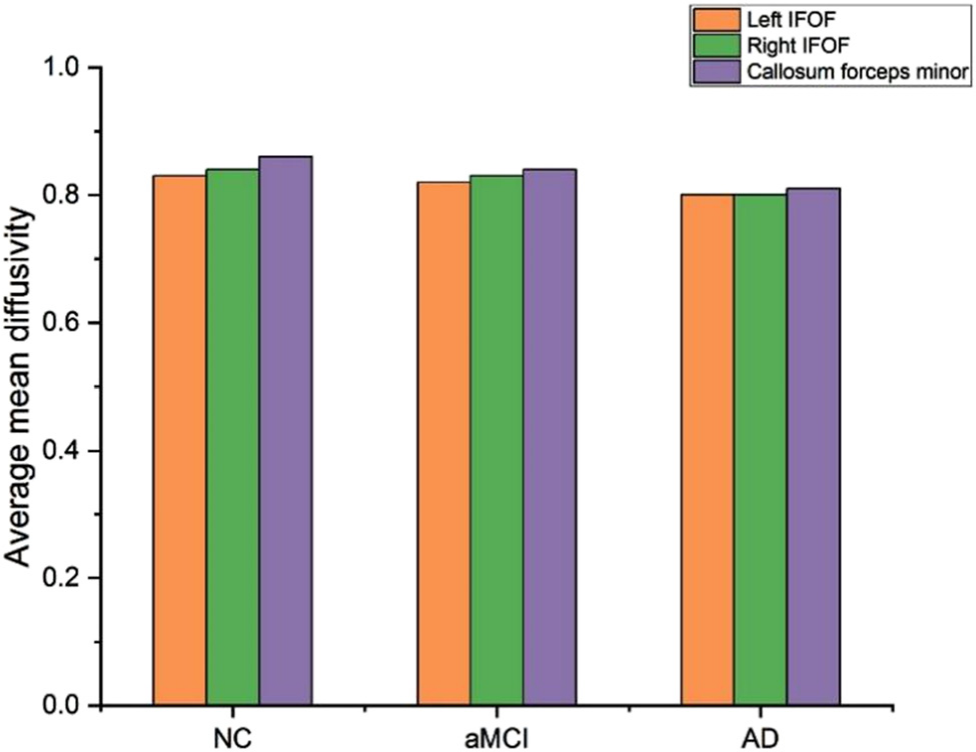
Average mean diffusivity.

Figure 9 displays the usual mean diffusivity profiles of the identified fiber tracts for the control and patient groups. The average tract MD profile and standard deviation for each group are shown. In the discovery dataset, the “left inferior frontal–occipital fasciculus (left IFOF), right inferior frontal–occipital fasciculus (right IFOF), and callosum forceps minor” regions show substantial changes between NCs, aMCI patients, and AD patients. The suggestive method used by NC is more precise than those used by aMCI and AD.

**Figure 10:**
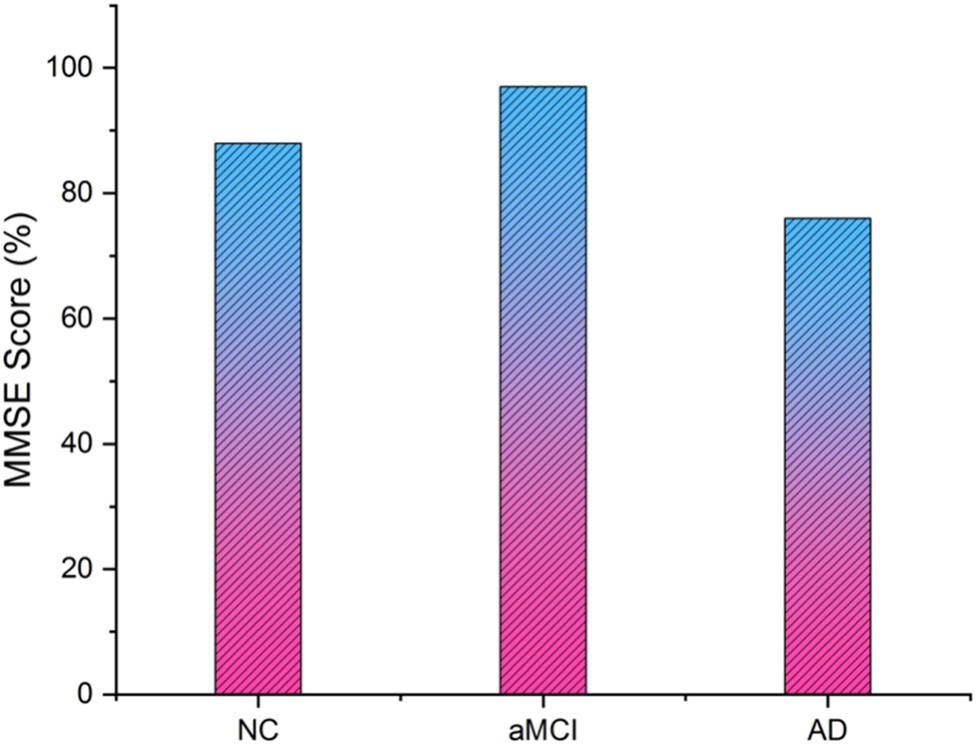
MMSE score.

Figure 10 depicts the MMSE scores. The MMSE is a frequently used test for detecting dementia and cognitive impairment. It is a quick survey or examination that evaluates several cognitive processes, including orientation, attention, memory, language, and visuospatial skills. The MMSE is often used to swiftly assess a person’s cognitive condition and spot any possible cognitive deficiencies in clinical settings. The MMSE is used as a screening tool to identify cognitive impairment rather than being intended to provide a conclusive diagnosis. A lower MMSE score may suggest possible cognitive impairments requiring more testing.

## 4 Discussion

In this research, we have suggested evaluating the impacts of all feasible combinations of various imaging modalities used for AD classification. Finding measures that may aid in precise early diagnosis, describe clinical states, or forecast illness development is one of the main goals of clinical neuroimaging. Compared to older people who are unaffected by MCI and have a 1–2% chance of dementia, amnestic MCI has a 20–40% risk of dementia [22]. Pathophysiological anomalies, tau- and A-related neurodegeneration, and cognitive impairment may emerge throughout the disease’s gradual course. For the sake of patient care and therapy research, early AD/MCI diagnosis is crucial. In Wang et al.’s [23] structural study that improved smooth classification, the classifier was a calibrated SVM, and the feature selection method was weighted smooth GL1/2. When GL1/2 reduces the number of intra-group and inner-group traits, group weights may further boost the effectiveness of the model. By including the calibrated hinge function, calibrated support vector machine may improve the speed and stability of the model. To assess the diagnostic value of microstructural integrity of extensive fascicular collections in differentiating “PSP-RS from PSP-P and AD,” we integrate proven probabilistic tractography with previously published DTI and dimensional measurements of subcortical brain structures [24]. Hellstrøm et al. [25] examines how APOE 4 interacts with brain morphometry and DTI to predict cerebral aging. Since DTI is sensitive to microstructural WM anomalies missed by traditional volumetric approaches, it may help in finding early in vivo biomarkers. We used two separate datasets to assess the integrity of whole-brain WM structures in patients with AD and moderate cognitive impairment. To our knowledge, we are the first to use a newly developed approach termed AFQ.

Furthermore, this study is the first to use independent cross-validation to test the efficacy of early AD identification based on the whole-brain WM architecture. Evidence of broad structural abnormalities in AD/aMCI can be consistently seen in patterns related to disease severity and impairment. A quantifiable imaging biomarker for AD/aMCI is possible because successful classification results are achieved using the most widely used machine learning SVM algorithm based on imaging characteristics. This in-depth investigation of brain abnormalities has important implications for understanding Alzheimer’s and other diseases affecting the WM. These methods might be used for AD/aMCI since they were precise and simple to replicate.

## 5 Conclusion

This experiment’s research assessed the effectiveness of integrating three imaging techniques often used for AD classification issues. The MM’s findings revealed the unexpected possibility that MR-DTI data fusion might enhance classification performance. In AD/aMCI, we found many tracts with aberrant and repeatable microstructural WM characteristics. According to the findings of the exploratory categorization, the examined diffusion measurements might be used as imaging biomarkers for AD. Some measures discussed in this study were accuracy, specificity, and sensitivity. In the discovery data, the suggested MM + MR-DTI achieved a 98% accuracy rate, 95% specificity, and 99% sensitivity; in the replication data, these figures dropped to 95% accuracy, 97% specificity, and 96% sensitivity. When compared to standard practice, the proposed approach fares well.

Additionally, these findings imply that, in the future, it would be able to diagnose AD only based on sMRI and DTI imaging data. In contrast to MRI and DTI, the patient will not be exposed to radiation, and the long acquisition methods of the MM will cause them less pain. This is important for elderly individuals with cognitive impairment. Additionally, because radioactive isotopes are not needed for developing, maintaining, or acquiring DTI, it is a less costly method. And last, using MRI and DTI pictures requires one piece of equipment.
